# XACML for Mobility (XACML4M)—An Access Control Framework for Connected Vehicles

**DOI:** 10.3390/s23041763

**Published:** 2023-02-04

**Authors:** Ashish Ashutosh, Armin Gerl, Simon Wagner, Lionel Brunie, Harald Kosch

**Affiliations:** 1 INSA Lyon, CNRS, LIRIS UMR 5205, 69100 Lyon, France; 2 Chair of Distributed Information Systems, Faculty of Computer Science and Mathematics, University of Passau, 94032 Passau, Germany

**Keywords:** access control, connected vehicles, time, location, frequency, ABAC, XACML, V2X

## Abstract

The automotive industry is experiencing a transformation with the rapid integration of software-based systems inside vehicles, which are complex systems with multiple sensors. The use of vehicle sensor data has enabled vehicles to communicate with other entities in the connected vehicle ecosystem, such as the cloud, road infrastructure, other vehicles, pedestrians, and smart grids, using either cellular or wireless networks. This vehicle data are distributed, private, and vulnerable, which can compromise the safety and security of vehicles and their passengers. It is therefore necessary to design an access control mechanism around the vehicle data’s unique attributes and distributed nature. Since connected vehicles operate in a highly dynamic environment, it is important to consider context information such as *location, time, and frequency* when designing a fine-grained access control mechanism. This leads to our research question: How can Attribute-Based Access Control (ABAC) fulfill connected vehicle requirements of *Signal Access Control (SAC), Time-Based Access Control (TBAC), Location-Based Access Control (LBAC), and Frequency-Based Access Control (FBAC)*? To address the issue, we propose a data flow model based on Attribute-Based Access Control (ABAC) called *eXtensible Access Control Markup Language for Mobility (XACML4M)*. XACML4M adds additional components to the standard eXtensible Access Control Markup Language (XACML) to satisfy the identified requirements of SAC, TBAC, LBAC, and FBAC in connected vehicles. Specifically, these are: *Vehicle Data Environment (VDE) integrated with Policy Enforcement Point (PEP), Time Extensions, GeoLocation Provider, Polling Frequency Provider, and Access Log Service.* We implement a prototype based on these four requirements on a Raspberry Pi 4 and present a proof-of-concept for a real-world use case. We then perform a functional evaluation based on the authorization policies to validate the XACML4M data flow model. Finally, we conclude that our proposed XACML4M data flow model can fulfill all four of our identified requirements for connected vehicles.

## 1. Introduction

The ever-present process of digitization has had a significant impact on the automotive sector. Today’s vehicles contain a growing number of tightly integrated systems, each consisting of a multitude of digital components such as engine management, telematics, and infotainment units. The advancement in automotive engineering has allowed vehicle sensor data to be collected, monitored, logged, analyzed, and monetized. Examples of this include the latitude, longitude, and speed of a vehicle. These data are then used to provide additional functionalities and services, such as autonomous driving, monitoring of vehicle health, and location-based restaurant recommendations. Essentially, the diverse array of services have access to personal data of the vehicle’s owner, which must be protected [1].

Numerous challenges arise with connected vehicles’ expanding connectivity, data storage, and feature set. First, ensuring the physical safety of passengers has been shown to be a non-trivial task. Notably, Miller, and Valasek [2] exposed safety issues in the 2014 Jeep Cherokee by injecting malicious messages into its physical systems. This enabled them to remotely engage brakes, accelerate, and even steer the vehicle, thereby compromising security and safety.

Second, the collection of vehicle data poses security and privacy concerns. Pesé et al. [3] categorize privacy-related attacks into driver fingerprinting, location inferencing, and analysis of driving behavior. They also identify *location, current speed, and steering wheel angle* sensor data as the most vulnerable to attacks. Finally, Krontiris et al. [4] discuss emerging privacy-related challenges in autonomous vehicles, such as the legal and ethical considerations while processing personal data, and usage of data minimization and retention methods.

To address the issues with security, privacy, and safety in the automotive industry, access control mechanisms are required. Designing an access control system for connected vehicles (CVs) is challenging because they operate in a dynamic environment in which the context of the vehicle is important when sharing (private) vehicle data. For instance, information about a vehicle’s location should be concealed to prevent an attacker from tracking the places that a person frequents. If exposed, these data could reveal sensitive information such as the person’s place of work and residence. Here, the location, time, and frequency of access are critical aspects of a potential access control system.

This led us to identify four access control requirements—*signal access, time-based access, location-based access, and frequency-based access*—that are necessary to consider for the automotive industry when designing an access control system. Indeed, access control has been thoroughly investigated in other domains such as health care, banking and finance. However, in a unique dynamic setting such as the connected vehicles environment, standard access control mechanisms are still being explored [5,6,7,8]. Furthermore, to the best of our knowledge, no automotive access control mechanism featured in literature has taken all four of our automotive requirements into account.

Thus, the primary motivation for our work was the lack of standard access control mechanisms for connected vehicles and the vulnerability of their sensor data. Based on our requirements, this paper aims to answer the following research question (RQ):

- *RQ: How can Attribute-Based Access Control (ABAC) fulfill connected vehicle requirements of signal access, time-based access, location-based access and frequency-based access control?*

To answer the *RQ*, we analyze the requirements and related work for access control in vehicles, in which we identify a research gap. We suggest an access control data flow model built on the ABAC-based eXtensible Access Control Markup Language (XACML) to fill this gap. The proposed data flow model is then validated using a functional evaluation based on authorization policies.

Note that we consider the following constraints for our work presented in this paper:

- We focus on the software residing in the application layer as opposed to the hardware layer inside of a vehicle;
- We do not consider physical access control of the vehicle and focus only on software-based access control mechanisms.

The paper is organized as follows: Section 2 provides a brief overview of terminology and concepts related to connected vehicles to elucidate the context. Then, Section 3 presents a use case that helps to derive the automotive requirements in Section 4. Section 5 presents related work on access control specific to the four identified requirements in automotive. Next, Section 6 presents our XACML4M data flow model, designed methodically to meet the identified requirements. Section 7 provides an evaluation of a proof-of-concept implementation of the XACML4M data flow model. Finally, we present our conclusion and propose future work in Section 8.

## 2. Background

Prior to discussing our use case and proposed model in later sections, automotive terminologies, vehicle communication mediums, and the ABAC access control model are introduced.

### 2.1. Automotive Terminology

**Definition** **1** (Connected Vehicles). *“Connected Vehicles (CVs) are those [vehicles] equipped with advanced communication technologies that allow the exchange of information, through different communication channels, between the various elements of the transport system [9]”.*

This definition is rather broad as it does not explicitly specify neither the communication technologies used, nor the elements of the transport system through which information is being exchanged. Thus, a concrete overview of different communication channels is given in Section 2.2.

Modern vehicles consist of many digital subsystems that enable them to function. Such subsystems comprise of three main components: *sensors, actuators and Electronic Control Units (ECUs)*. It is estimated that modern cars roughly contain between 60 to 100 *sensors* [10], with the industry expecting the number to reach 200 throughout the next years as additional automotive trends such as autonomous driving are being implemented [11].

Various types of *sensors* are installed in vehicles, e.g., wheel speed, acceleration, steering, outdoor temperature along with various engine properties such as engine speed or fuel–air ratio [12]. Within the context of self-driving cars, more complex sensor types are being introduced, including cameras, ultrasonic, Light Detection and Ranging (LIDAR), and Radio Detection and Ranging (RADAR) [13].

**Definition** **2** (Actuators). *“Actuators are required in mechatronic systems for the generation of movements or the application of forces. The term [...] includes all types of output elements for movements and forces ([14], translated)”.*

Actuators are present within engines to regulate various functions such as fuel supply, air control, or gas re-circulation for ensuring smooth engine operation. Both *sensors* and *actuators* are connected to and controlled by *Electronic Control Units (ECUs)*.

**Definition** **3** (Electronic Control Units-ECUs). *“ECUs are embedded computers that monitor automotive systems via sensors, control the vehicle via actuators, or report vehicle data [15]”.*

Depending on the size and the feature set of the vehicle, it may contain roughly around 70 [16] to more than 100 interconnected ECUs [17]. Next, we define vehicle telematics that collects sensor data to provide safety and enhance the driving experience.

**Definition** **4** (Telematics). *“The term “telematics” is composed of the words “telecommunication” and “informatics”. Le et al. define telematics systems as “[...] systems that provide information and support entertainment as well as financial transactions; and complex Advanced Driver Assistance Systems (ADAS) that aim to turn vehicles into intelligent systems, improve safety, and enhance [the] driving experience” [15]”.*

After presenting the basic automotive terminologies, next, we look into different entities that a vehicle communicates in a connected vehicle ecosystem and how the data generated by *ECUs* and *telematics* are exchanged.

### 2.2. Vehicle Communication

Modern vehicles are not self-contained anymore. Throughout the last decades, various communication interfaces have been adopted that allow for the exchange of data with the outside world. External communication between vehicles and other entities is generally referred to as V2X (“Vehicle to Everything”). An overview is given below:

- **Vehicle to Device (V2D)**: *V2D* communication describes data exchange with any type of electronic device, for example, a smartphone running a vehicle application. The app may allow, for instance, HVAC remote control or charge monitoring for electric vehicles.
- **Vehicle to Vehicle (V2V)**: *V2V* is typically mentioned alongside *Vehicular Ad-Hoc Networks (VANETs)*. The key idea behind V2V is that vehicles on the road dynamically establish a wireless mesh network over which data can be exchanged. Some possible applications are reporting route obstacles in a decentralized manner by traffic information systems, collision warning systems, and adaptive cruise control [18].
- **Vehicle to Infrastructure (V2I)**: *V2I* refers to the exchange of information between vehicles and road infrastructure, commonly referred to as Road-Side Units (RSUs). For example, road markings, traffic lights, signs as well as intersections.
- **Vehicle to Cloud (V2C)**: In *V2C*, vehicles communicate with different cloud-based services, usually via applications installed inside the vehicle, for example, to receive Over-The-Air (OTA) updates and share diagnostic reports with the vehicle manufacturers.
- **Vehicle to Pedestrian (V2P)**: *V2P* primarily aims to enhance safety for pedestrians. Example applications are pedestrian detection and warning systems. This not only issues warnings to drivers, but also to pedestrians who are in the vicinity of the vehicle.
- **Vehicle to Grid (V2G)**: *V2G* is concerned with data and energy exchange between electric vehicles and the smart grid. This is used, for example, to integrate electric vehicles into the power grid as an energy storage option, aiding grid stabilization as well as allowing the bridging of power outages in the context of the renewable energy transition.

V2X communication provides an understanding of different interactions occurring in a connected vehicle ecosystem and these interactions also involve the exchange of vulnerable sensor data leaving the vehicle. Different types of V2X communication in a connected vehicle environment bring a unique set of challenges and require a carefully designed access control framework. For example, Albouq and Fredericks [19] designed an ABAC-based framework for secure *V2I* communication between RSUs and service providers. In this paper, we focus on V2C communication for which a corresponding use case is defined in Section 3. Next, we present briefly the ABAC model which is a fine-grained and distributed access control model and is the basis for our proposed solution.

### 2.3. Attribute Based Access Control Model

A high-level description of ABAC is provided by the National Institute of Standards and Technology (NIST):

“[ABAC is] an access control method where subject [access] requests to perform [actions] on [resources] are granted or denied based on assigned attributes of the subject, assigned attributes of the [resource], environment conditions, and a set of policies that are specified in terms of those attributes and conditions.”

([20], with adapted terminology)

Typical ABAC systems follow a distributed concern-separating approach in that the access control mechanism is split across multiple entities. The NIST guide to ABAC [20] highlights four important functional entities as mentioned below:

- **Policy Enforcement Point (PEP)**: Enforces access control decisions. Upon an access request, the PEP forwards this request to the Policy Decision Point (PDP). Depending on the PDP’s decision, access to the requested resource is either granted or denied.
- **Policy Decision Point (PDP)**: Evaluates access control decisions. The PDP computes whether to allow or deny an access request based on the configured policies in the access control system and the attributes of subjects, resources, and environment.
- **Policy Information Point (PIP)**: Resolves required attributes during policy evaluation.
- **Policy Administration Point (PAP)**: Provides an interface for policy management.

For our work, we use the eXtensible Access Control Markup Language (XACML), which is a standardized policy language schema for ABAC. It is currently available in version 3.0 [21] and is based on the eXtensible Markup Language (XML) data format that has been proposed by the Organization for Advancement of Structured Information Standards (OASIS).

After drawing a basic overview of the entities involved in a connected vehicle ecosystem and the interactions between them, next, we present a realistic use case which is then used to identify automotive access control requirements.

## 3. Use Case

In this section, we present our use case which is later used to derive our requirements specific to the automotive domain.

Consider Alice, who recently purchased a new smart car and by law, every car needs motor insurance. Thus, she signs up with a car insurance provider called *SmartSurance*. *SmartSurance* offers personalized discounts based on her driving style, but also requires her to install an application in the infotainment system (her car’s software platform), which requires monitoring data from several sensors to provide the personal discount service.

**Figure 1:**
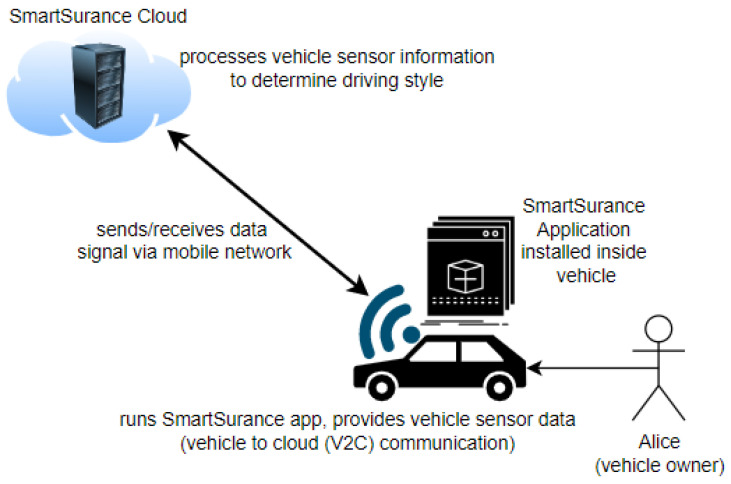
Use Case—Vehicle-to-Cloud (V2C) communication between the SmartSurance application installed in the vehicle and the SmartSurance service in the cloud.

*SmartSurance* requires access to *current location, vehicle speed, engine RPM, and throttle position* of the vehicle. The application requires *one sample of these sensor values every second*. The collected sensor data are eventually transferred to the cloud where they are further processed by the insurance provider (see Figure 1). Alice’s insurance tariff requires vehicle data to be collected only when she travels routes outside her hometown, which is assumed to be the town of Haiden in Bavaria, Germany. Furthermore, she exempts herself from data collection during off-peak hours, which are assumed to be from *Monday to Friday* between *5:05 PM and 8 PM* for privacy reasons.

After describing our use case, next, we derive the requirements that are applicable to the automotive domain.

## 4. Requirements

This section introduces the four functional requirements specific to automotive that were identified in cooperation with the World Wide Web Consortium (W3C) Automotive Working Group [22] during our regular discussions. W3C automotive group is a group of experts from different stakeholders (vehicle manufacturers, suppliers, solution providers and researchers) who together with the Connected Vehicle Systems Alliance (COVESA) work on developing standards within the automotive domain.

Accounting for the vast amount of potential use cases in a connected vehicle ecosystem and the associated challenges with regard to security and privacy, these requirements were defined and derived from our previous paper [23]. The four functional requirements are *Signal Access Control (SAC), Location-Based Access Control (LBAC), Time-Based Access Control(TBAC) and Frequency-Based Access Control (FBAC)* specific to the automotive use case described in Section 3. For each requirement, we present its definition, rationale, and a sample use case to show its access control message flow.

### 4.1. Signal Access Control (SAC)

**Figure 2:**
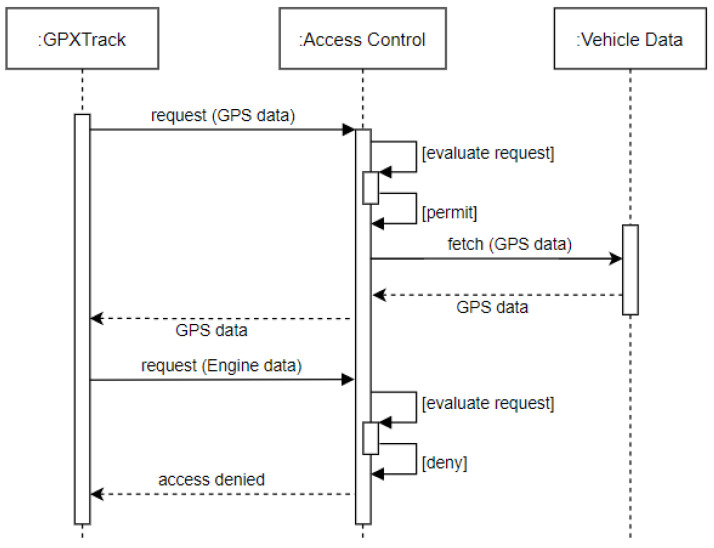
Access Control Flow for Signal Access Control (SAC)—GPS data access is authorized and engine data access is restricted.

- *Requirement Definition*: This is a primitive access control requirement, wherein access to vehicle sensor data should be granted only to authorized entities. Arbitrary access requests must be restricted.
- *Requirement Rationale*: This requirement is fundamental from a safety and security standpoint. For instance, an application installed for analyzing driving behavior by collecting sensor data such as average speed, Rotations Per Minute (RPM) and maximum speed must not be able to trigger an emergency stop while the vehicle is in motion by writing corresponding values to the hard braking sensor as this would put the driver and other individuals on road at risk.
- *Use Case*: Consider Alice, who has installed a GPS location tracker application called *GPXTrack* in her vehicle. Alice uses this application to record geolocation data for roads that have not been mapped onto *OpenStreetMap*, a map application. GPXTrack requires access to the latitude and longitude sensor data (GPS) of the vehicle to which it has been authorized access. It must not be allowed to arbitrarily access other vehicular sensor data (see Figure 2).

### 4.2. Time-Based Access Control (TBAC)

**Figure 3:**
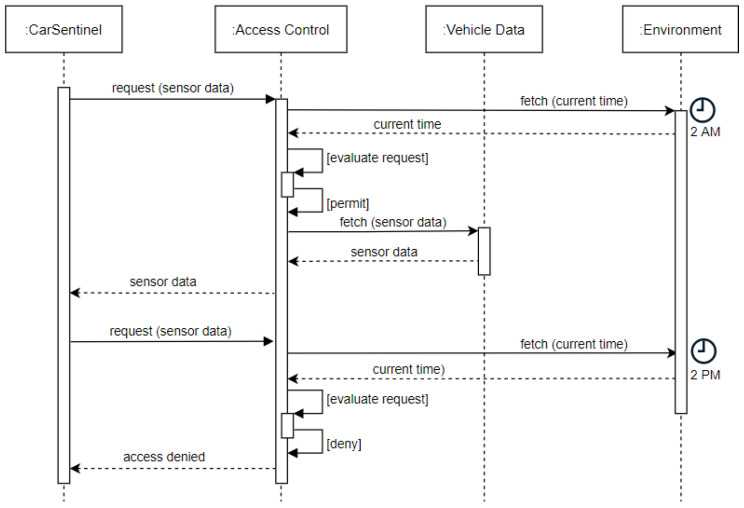
Access Control Flow for Time-Based Access Control (TBAC)—Access request at 2 AM, which is within the allowed time period of 8 PM to 8 AM, is granted, otherwise, it is denied.

- *Requirement Definition*: An access control mechanism should consider *time periods* for granular access control enforcement when evaluating an access request.
- *Requirement Rationale*: Vehicles in a connected vehicle ecosystem are a data source that helps mitigate challenges such as traffic management, threat alerts, and accident warnings, among others. A user willing to share their vehicle data and help alleviate such challenges might have some restrictions based on time. For example, they might not be willing to share their data (on weekends) for privacy or security reasons because they go hiking on weekends, and exposing the time duration of their trip could make their home vulnerable.
- *Use Case*: Consider Alice who installs an application called *CarSentinel*, an anti-theft app, in her vehicle. *CarSentinel* requires access to vehicle sensors—microphones, cameras, and microwave radar proximity sensors to determine the surroundings of the vehicle. Alice does not want *CarSentinel* to be active during the daytime as she uses it to get to work. Consequently, the app must be able to access the vehicle sensors only at night, which is assumed to be between 8 PM and 8 AM (see Figure 3). To evaluate the access request received by *CarSentinel*, the current time needs to be inferred by the access control mechanism from the environment. If vehicle sensor data are accessed within the aforementioned time interval (8 PM to 8 AM), then access is granted, otherwise, it is denied. Note that there might be additional constraints set by Alice based on the day(s) of the week, for example, weekends when her vehicle is parked in the garage and she does not want her vehicle data to be accessed.

### 4.3. Location-Based Access Control (LBAC)

**Figure 4:**
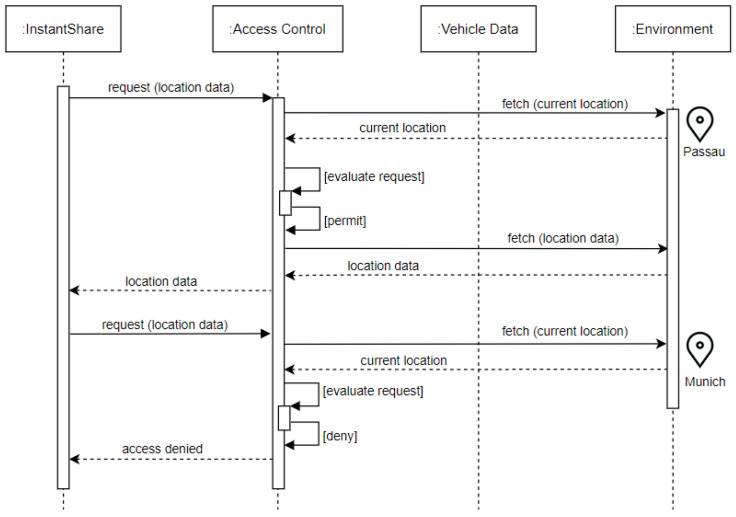
Access Control Flow for Location-Based Access Control (LBAC)—Access request within the city limits of Passau is granted, otherwise, it is denied.

- *Requirement Definition*: An access control mechanism should consider the *location* of the vehicle when evaluating an access request. This is relevant, especially in a connected vehicle ecosystem where location technologies have become more precise. Similar to time, location-based access should be enforced for allowing the user to share their vehicle data in a flexible setting based on their convenience.
- *Requirement Rationale*: The reasoning behind this requirement is very similar to time-based access. A user might be willing to share location but under certain conditions might not because of security or privacy concerns.
- *Use Case*: Consider Bob who is interested in sharing his vehicle when it is not in use. Bob installs an application *InstantShare* that requires access to the location of his car (latitude and longitude) regularly which is then sent to its servers in the cloud for processing and evaluating currently available vehicles for sharing. Since Bob lives in a well-connected city called Passau (Germany), he decides to use local transport when in Passau. This allows him to share his vehicle with people who want to use his vehicle within Passau city limits for some additional money. Sometimes, he travels out of town for work or personal reasons with his vehicle and does not want to share his vehicle, otherwise, he would be stranded. As such, *InstantShare* must only be able to access his car’s location data as long as it is located within the city limits of Passau (see Figure 4).

### 4.4. Frequency-Based Access Control (FBAC)

**Figure 5:**
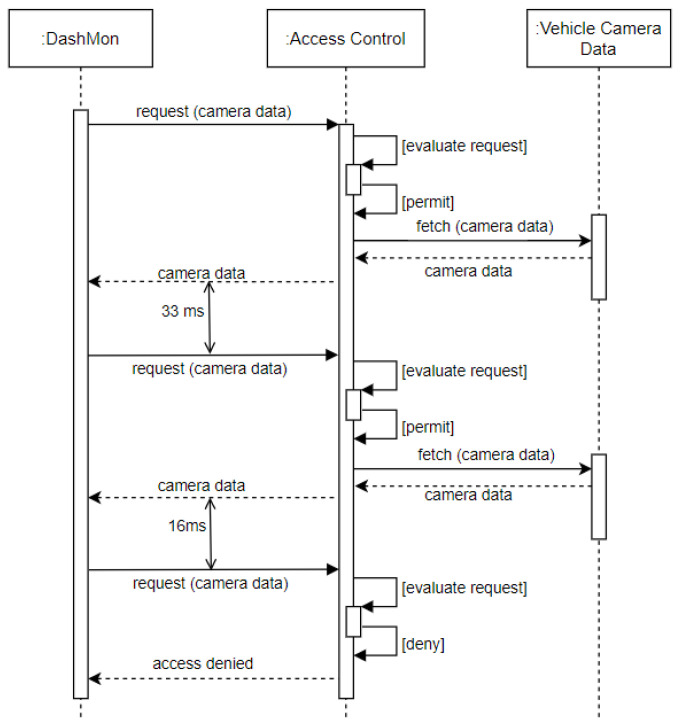
Access Control Flow for Frequency-Based Access Control (FBAC)—Access request frequency when >=33 ms is granted, otherwise, it is denied.

- *Requirement Definition*: An access control mechanism should consider the *frequency* of requests that can be made by an authorized entity. A vehicle is a safety-critical system and processing multiple requests from a single application may overtax the ECUs which could then restrict their ability to perform critical tasks.
- *Requirement Rationale*: Restricting the frequency of access to vehicle sensor data by an application prevents overloading communication channels (e.g., Controller Area Network (CAN) bus) with incoming signals. If not limited, this could raise a safety issue, particularly when critical components such as the engine, brakes, or steering are affected. Additionally, there are instances where users may wish to restrict the frequency of data access for privacy reasons. For example, it may be undesirable for vehicle owners to share their location data within close-spaced intervals as this would provide thorough information about their motion profile.
- *Use Case*: Consider Alice, who opted to install an application called *DashMon* in her vehicle, which is an application that periodically accesses her vehicle’s front and rear cameras to take photos for *dash cam* functionality. To not overtax the underlying communication channels, *DashMon* is only allowed to take 30 photos per second, i.e., photos may be taken at a *33ms* time interval (see Figure 5).

To summarize, an access control mechanism for a connected vehicle ecosystem must consider environmental parameters such as location and time. This is necessary, especially in V2X connectivity where sensor data are vulnerable when they are shared with the surroundings such as the cloud, vehicles, and infrastructure. Additionally, with multiple entities requesting access, the frequency of access also needs to be limited to avoid overtaxing the resources inside a vehicle.

## 5. Related Work

The evolution of software systems in vehicles and the exchange of sensor and personal data in a V2X setting has led to more research being conducted on access control mechanisms for automobiles. In this section, we look into the scientific literature for different access control mechanisms in the automotive industry and compare them to our identified requirements (see Section 4). Additionally, in each related work, we also observe for the type of access control model used.

### 5.1. Signal Access Control (SAC)

SAC is defined as granting access only to authorized entities which is the most basic requirement for any access control mechanism. Thus, it is reflected in all of our related work references with the exception of Kchaou et al.’s proposal [24], as the proposed model focuses on access to globally available services, i.e., weather reports or road information rather than individual vehicle sensor data.

### 5.2. Frequency-Based Access Control (FBAC)

FBAC limits frequency of access requests that can be made by an authorized entity. Rumez et al. [5] propose a framework for distributed automotive based on ABAC model that operates close to the hardware level. They present an architecture model where they modify the CAN bus payload to include attributes (within the CAN payload) for fine-grained access control to protect domain controllers and ECUs in the vehicle [5]. They briefly mention a rate-limiting technique at the Policy Enforcement Point (PEP), potentially indicating that some kind of frequency based access control is supported. However, this was not further elaborated.

Another work by Kim et al. [8] integrated fine-grained ABAC directly into the *service* layer of AUtomotive Open System Architecture (AUTOSAR) [25]. Their contribution was theoretical, using cryptography-based routines for access control. Environment variables such as location and time were not considered, although frequency-based access was mentioned briefly. Plappert et al. [26] also propose a simple cryptography-based access control where only entities in possession of corresponding *keys* can decrypt the data. They take into account the frequency of access using log storage in their system model.

### 5.3. Time-Based Access Control (TBAC)

TBAC requires fine-grained access where *time periods* are considered when evaluating an access request. Zhang et al. [27] developed an access control framework called *AC4AV* that uniquely distinguished between real-time and historical data and supported three different access control models, i.e., Discretionary Access Control (DAC), Identity-Based Access Control (IBAC), and Attribute-Based Access Control (ABAC). They used different component names but their functionality was similar to that of ABAC framework. TBAC appears to be supported as mentioned in their paper, although no concrete policy examples were provided.

In another work by Alsarra et al. [28], they propose a theoretical access control model for vehicular networks based on RBAC. Their proposed OpenRBAC model sets itself apart from other models with its distinction between open and closed domains with automotive belonging to an open domain where roles within RBAC cannot be defined uniformly. If the role of the subject is unknown, then a score function is used to calculate and assume a respective role to the subject. A formal definition was provided for OpenRBAC and TBAC may be realized through relative and absolute context attributes.

### 5.4. Location-Based Access Control (LBAC)

LBAC is defined as an access control mechanism where the *location* of the vehicle is considered when evaluating an access request. Gupta et al. [6] propose a Vehicle to Vehicle (V2V)-based novel notion of *groups* where vehicles are assigned to groups based on their dynamic attributes, such as *location or speed*. Location-based access control using ABAC was a key feature of their CV-ABAC_G_ model. However, CV-ABAC_G_ is an access control that, in its current state, depends entirely on a single cloud provider and its proprietary technologies.

Furthermore, location-based access was also considered in [26,27,28], although none of them provided any reference policy to compare our work. For example, [28] only provides a formal definition of their model and [27] stores access logs, policies, and configuration files but does not provide access to them.

### 5.5. Discussion

The access control models that have been proposed in this literature review are summarized in Table 1. We compare the work in the literature with our identified requirements and also observe the type of access control model used.

SAC, as the most fundamental requirement, is reflected in eight of the nine works mentioned. FBAC, TBAC, and LBAC are reflected sporadically, and none of the works except [5] provided any access to their implementation. This made it challenging to experiment with their proposed models. Additionally, none of them were found to consider all four of our requirements. Seven out of the nine works that were surveyed employed some form of ABAC instantiation (see Table 1). As a result, it confirms our previous findings [23] that ABAC is a suitable model for automotive access control requirements.

Particularly noticeable is the presence of XACML [21] which is an ABAC standard and used in two of the ABAC-based proposals ([8,26]) to define policies. XACML is well established with good documentation and it has several open-source implementations to aid development [29,30]. We have already established (see Table 1) that no ABAC-based access control model satisfies all four requirements; thus, we have to decide on an ABAC model that is extensible and well known in the literature.

We have seen in the related work presented that XACML is a common standard used to build on and is also extensible. Abbreviated Language For Authorization (ALFA) is an alternative policy language to XACML. In our use case, where we want to extend the functionality, both are equivalent because ALFA is a domain-specific language for a high-level description of XACML policies, and it is possible to perform a lossless round-trip translation from ALFA to XACML and vice versa [31]. Furthermore, to the best of our knowledge, no other ABAC-based model enables the required extensibility of our use case at an equivalent or better level than XACML or ALFA. Thus, we use the ABAC-based XACML [21] standard as the basis for our data flow model, *XACML4M* which we present in Section 6.

It has to be considered that the policy structure of XACML is complex [32]. Thus, it makes it challenging to use, especially for stakeholders and other individuals who are knowledgeable about security and privacy issues, but lack advanced technical skills. To aid the understanding of models such as XACML, multiple visual specification languages have been proposed in the literature [33,34,35,36]. Giordano et al. [34] propose an editor to visually define policies or security diagrams by retrieving the list of subject, action, and resource entities from the application server, which is then translated into XACML.

Additionally, Nergaard et al. [37] define a policy editor for XACML using *Scratch*. Heydon et al. [33] introduce a constraint language to specify security policies using simple boxes and arrows. Basin et al. [35] and Koch et al. [36] propose access control models using UML and graphs, respectively, for RBAC systems. In contrast to [33,34,35,36], for Layered Privacy Language (LPL), a visual representation is provided to define policies and also help users personalize them [38]. In this paper, we only focus on modifying the data flow model of XACML and not a visual representation of policy language because our objective is to evaluate the functionality and not the usage of XACML. We intend to evaluate the usage of modified XACML and use a visual specification language as a future step.

## 6. XACML for Mobility (XACML4M)

As shown in the previous Section 5, we use the XACML data flow model as the basis to propose *eXtensible Access Control Markup Language for Mobility (XACML4M)* to comply with the *SAC, TBAC, LBAC, FBAC* requirements. We first provide an overview of the model and then we specify the individual components methodically added to meet each requirement.

### 6.1. XACML4M

XACML standard establishes an architecture, a processing model, and a declarative fine-grained, attribute-based access control policy language that describes how to evaluate access requests in accordance with the rules set forth in policies [21]. When compared to literature work [5,6,8,19], they use XACML in its standard form without any modifications. They either use it to define policies and integrate it at the hardware layer within the automotive architecture, or to combine it with other automotive frameworks such as AUTOSAR [25]. Additionally, they do not consider the combination of our requirements—*location, time, and frequency*. The XACML data flow model in its original form is generic and does not satisfy the automotive-specific requirements [21].

In our modified data flow model called XACML4M based on XACML, we make the following changes:

- Add data types and functions to the XACML policy—We add dayOfWeek data type and dateTime-in-dayOfWeek-range function to account for TBAC; Geometry data type to define a polygon and urn:ogc:def:function:geoxacml:1.0:geometry-contains function to determine whether a point is contained inside the area that is enclosed by the polygon; a custom data type urn:tf:cyber:xacml:polling-frequency:time-since-last-access:ms to retrieve time since last access made by a requesting entity.
- Add components to the data flow model:
  – *Vehicle Data Environment (VDE) integrated with Policy Enforcement Point (PEP)*: VDE is a setup through which vehicle sensor data are accessed and PEP receives all incoming access requests from any application/service.
  – *Time Extensions*: Provides *time period* data type as an attribute for policy evaluation by PDP.
  – *GeoLocation Provider*: Provides geospatial data type as an attribute and also fetches the current location of the vehicle for policy evaluation by PDP.
  – *Polling Frequency Provider*: fetches the timestamp of the last access made by a requesting entity (an application/service).
  – *Access Log Service*: records the last successful access made by a requesting entity (an application/service).

**Figure 6:**
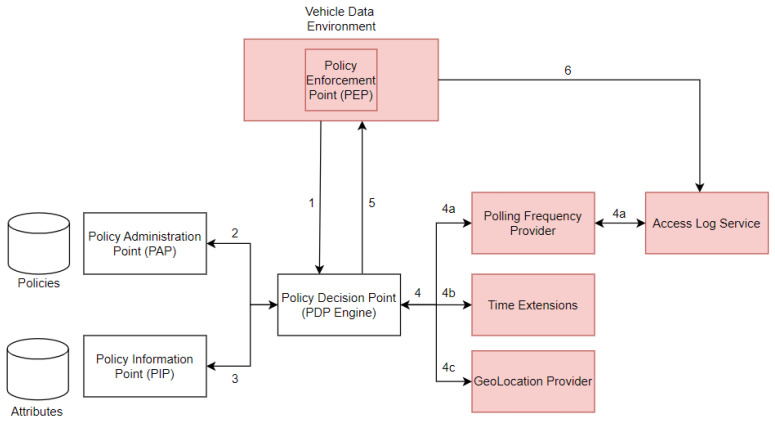
XACML4M Data Flow Model (based on XACML data flow model [21])—components added specifically to automotive requirements are highlighted.

Figure 6 presents the data flow model which includes *PDP, PIP, PAP, and PEP* ABAC components (see Section 2.3) and automotive-specific components *Polling Frequency Provider, Access Log Service, Time Extensions, GeoLocation Provider, and Vehicle Data Environment*. The numbers in Figure 6 show the sequence of message flows when an access control request is received by the PEP and processed by the PDP with the assistance of PAP, PIP, and automotive-specific components. For example, path *4a* is enabled when *FBAC* is required, path *4b* is enabled when *TBAC* is required, and path *4c* is enabled when *LBAC* is required during policy evaluation by PDP.

Next, we look into each new component added to the data flow model and describe how they satisfy each identified requirement.

#### 6.1.1. Vehicle Data Environment (VDE) with PEP

**Figure 7:**
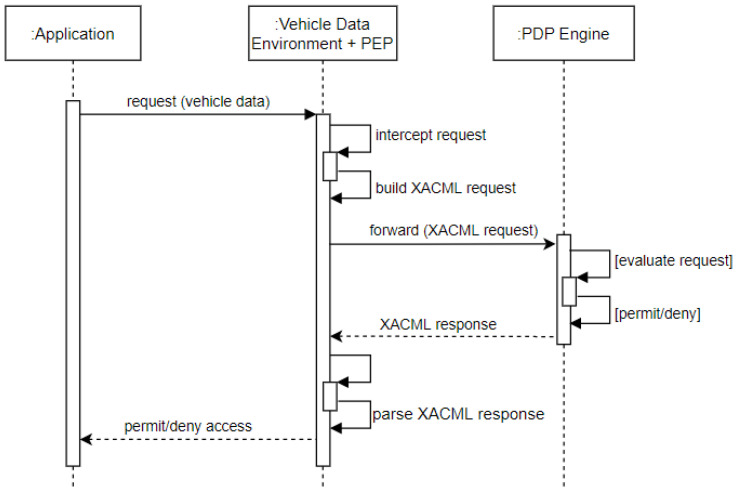
XACML4M access control enforcement message flow.

The Vehicle Data Environment (VDE) is a setup to access vehicle sensor data via *VDE REST* controllers. Note that it is possible to specify in VDE which sensor data needs to be protected by the access control mechanism. The PEP component which is integrated within the VDE intercepts all incoming access requests made to the VDE. Figure 7 shows the message sequence flow for incoming requests.

The XACML4M access control enforcement is facilitated in four steps:

1. **Request Assembly**: If sensor data are guarded by XACML access control, then the PEP intercepts the request and first builds an access request that adheres to the JSON profile of XACML [39]. An access request typically contains the following attributes:
  - Subject ID (String): The identifier of the requesting subject.
  - Action ID (String): The identifier of the requested action.
  - Resource ID (String): The identifier of the requested resource.
  Note that in our framework, the PEP can also include additional attributes such as current location (in case of LBAC requirement) or current time (in case of TBAC requirement) to the XACML request before submitting it to the PDP and thus aiding in its policy evaluation.
2. **Request Submission to PDP**: The resulting XACML request is submitted to the PDP component for access evaluation. Depending on the underlying setup, this component may be placed externally to the vehicle and can be configured accordingly. The PDP evaluates the incoming request against all policies currently in effect and responds with its decision.
3. **Response Evaluation**: After policy evaluation, the PDP’s response is received by the PEP. If its access decision is *Permit*, the sensor data access is granted. Otherwise, access is denied. Note that the PEP component heavily relies on aspect-oriented programming (AOP) which facilitates enabling/disabling access control for an entire set or subset of vehicle signal data.
4. **Obligation Fulfillment (optional)**: XACML provides the concept of obligations. In the case of our XACML4M framework, the *Obligations Service* is directly embedded into the PEP. Should an XACML response from PDP include obligations, they are executed before the sensor data are accessed. The business logic of all obligations is implemented as part of the PEP. A concrete example will be given in Section 6.1.5.

Summarizing, so far, this Section presented the XACML4M data flow model and highlighted the roles of each component. Then, we also detailed the XACML4M access control enforcement mechanism. Next, we address the implementation of each automotive access control requirement as outlined earlier (see Section 4), along with authorization policy examples that realize the previously introduced use cases (see Section 3).

#### 6.1.2. XACML4M Signal Access Control (SAC)

For the Signal Access Control (SAC) requirement, it is necessary to allow access only to the authorized resources with allowed actions to be performed on that resource. This can be achieved by specifying the triplets *subject, object, and action* in the authorization policy, and arbitrary signal access can be prevented. Signal access is the most basic of all the requirements and can be realized with means that are part of the XACML 3.0 specification.

Referring to the use case given in Section 4.1, it is assumed that Alice’s *GPXTrack* application is authenticated to the vehicle data environment and can access the GPS data via a simple HTTP GET operation on the /vehicle/location route. The corresponding authorization policy that prevents *GPXTrack* from accessing any resource other than the vehicle’s current location is depicted in Listing 1.

Listing 1 **XACML-Signal Access-the Subject (gpxtrack) is requesting Resource (vehicle/location) to perform Action (GET).**


<?xml version="1.0" encoding="UTF-8" standalone="yes"?>
<Policy
<!--Policy definition-->
        xmlns="urn:oasis:names:tc:xacml:3.0:core:schema:wd-17"
        Version="1.0" PolicyId="GPXTrackPolicy"
        RuleCombiningAlgId="urn:oasis:names:tc:xacml:3.0:rule-combining-algorithm:deny-unless-permit">
  <Target>
    <AnyOf>
      <AllOf>
        <Match MatchId="urn:oasis:names:tc:xacml:1.0:function:string-equal">
<!--Subject data type and value-->
        <AttributeValue DataType="http://www.w3.org/2001/XMLSchema#string">gpxtrack</AttributeValue>
          <AttributeDesignator
            Category="urn:oasis:names:tc:xacml:1.0:subject-category:access-subject"
            AttributeId="urn:oasis:names:tc:xacml:1.0:subject:subject-id" MustBePresent="true"
            DataType="http://www.w3.org/2001/XMLSchema#string"/>
        </Match>
        <Match MatchId="urn:oasis:names:tc:xacml:1.0:function:string-equal">
<!--Resource data type and value-->
          <AttributeValue DataType="http://www.w3.org/2001/XMLSchema#string">/vehicle/location</AttributeValue>
          <AttributeDesignator
            DataType="http://www.w3.org/2001/XMLSchema#string"
            AttributeId="urn:oasis:names:tc:xacml:1.0:resource:resource-id"
            Category="urn:oasis:names:tc:xacml:3.0:attribute-category:resource"
            MustBePresent="true"/>
        </Match>
        <Match MatchId="urn:oasis:names:tc:xacml:1.0:function:string-equal">
<!--Action data type and value-->
          <AttributeValue DataType="http://www.w3.org/2001/XMLSchema#string">GET</AttributeValue>
          <AttributeDesignator
            DataType="http://www.w3.org/2001/XMLSchema#string"
            AttributeId="urn:oasis:names:tc:xacml:1.0:action:action-id"
            Category="urn:oasis:names:tc:xacml:3.0:attribute-category:action" MustBePresent="true" />
        </Match>
      </AllOf>
    </AnyOf>
  </Target>
  <Rule Effect="Permit" RuleId="AllowGPS"></Rule>
</Policy>

The <Target> clause in the authorization policy defines the access requests to which the policy would be applied and evaluated. In Listing 1, it makes sure that the policy is only applied to GET requests made by *GPXTrack* to /vehicle/location resource. The policy further defines a single *rule* with id AllowGPS that does not specify any <Condition>. As such, the *rule* automatically enters into effect and permits access. In this use case, any request to access a different resource made by *GPXTrack* yields a *Not Applicable* policy evaluation result because the policy’s <Target> clause would not be fulfilled. After the evaluation of the authorization policy by the PDP, if *permit* was returned to the PEP, then it activates the function at the endpoint /vehicle/location which returns the *location* data. Thus, the requirement SA is fulfilled.

#### 6.1.3. XACML4M Time-Based Access Control (TBAC)

In this requirement, data types were needed to consider not only *time range*, but also the *days of the week* to provide fine-grained access control. The time-based access control requirement is partially realizable using functionality present in core XACML 3.0. Specifically, it is possible to define time ranges—which do not account for days of the week—without any further extensions to the data flow model using the existing XMLSchema#time datatype, along with the already supplied time-in-range function. Accounting for the use case previously introduced in Section 4.2, where access shall only be granted between 8 pm and 8 am, the following <Condition>, as depicted in Listing 2, only evaluates to true if and only if the current time of day lies within this time frame, assuming that the timezone is GMT+2.

Listing 2 **XACML-Time-based Access Control-only Time Condition can be specified using default XACML 3.0.**


<Condition>
<!--Function "time-in-range" - to check time range-->
  <Apply FunctionId="urn:oasis:names:tc:xacml:2.0:function:time-in-range">
    <Apply FunctionId="urn:oasis:names:tc:xacml:1.0:function:time-one-and-only">
      <AttributeDesignator
        DataType="http://www.w3.org/2001/XMLSchema#time"
<!Parameter 1 is the current time passed to Function "dateTime-in-dayOfWeek-range"-->
        AttributeId="urn:oasis:names:tc:xacml:1.0:environment:current-time"
        Category="urn:oasis:names:tc:xacml:3.0:attribute-category:environment"
        MustBePresent="true"/>
    </Apply>
    <AttributeValue
<!Parameter 2 is the start time passed to Function "time-in-range"-->
    DataType="http://www.w3.org/2001/XMLSchema#time">20:00:00+02:00</AttributeValue>
    <AttributeValue
<!Parameter 3 is the end time passed to Function "time-in-range"-->
    DataType="http://www.w3.org/2001/XMLSchema#time">08:00:00+02:00</AttributeValue>
  </Apply>
</Condition>

Additional functionality was added for the time-based access control data flow model (see Figure 6—*Time Extensions Component*) in the XACML4M framework, wherein *days of the week* can also be checked as a <Condition> along with *time* as part of the authorization policy description, as shown in Listing 3. This extension was based on XACML 3.0 Time Extensions Standard [40], which is currently still in the draft stage.

Listing 3 **XACML-Time-based Access Control—our extension (integrating XACML 3.0 Time Extensions) allows Time and Weekday Condition to be verified—it provides fine-grained access control.**


<Condition>
<!--Function "dateTime-in-dayOfWeek-range" - to check time range and day of Week-->
  <Apply FunctionId="urn:oasis:names:tc:xacml:3.0:function:dateTime-in-dayOfWeek-range">
    <Apply FunctionId="urn:oasis:names:tc:xacml:1.0:function:dateTime-one-and-only">
      <AttributeDesignator
        Category="urn:oasis:names:tc:xacml:3.0:attribute-category:environment"
<!Parameter 1 is the current time passed to Function "dateTime-in-dayOfWeek-range"-->
        AttributeId="urn:oasis:names:tc:xacml:1.0:environment:current-dateTime"
        DataType="http://www.w3.org/2001/XMLSchema#dateTime"
        MustBePresent="false"/>
    </Apply>
<!Parameter 2 is start time/day range to Function "dateTime-in-dayOfWeek-range"-->
<!1 - Monday, 2 - Tuesday, 3 - Wednesday ...."-->
    <AttributeValue DataType="urn:oasis:names:tc:xacml:3.0:data-type:dayOfWeek">1+02:00</AttributeValue>
<!Parameter 3 is end time/day range to Function "dateTime-in-dayOfWeek-range"-->
    <AttributeValue DataType="urn:oasis:names:tc:xacml:3.0:data-type:dayOfWeek">5+02:00</AttributeValue>
 </Apply>
</Condition>

#### 6.1.4. XACML4M Location-Based Access Control (LBAC)

Considering the use case in Section 4.3 where Bob wants to share his vehicle only when the vehicle is located in the city limits of Passau, Germany, we would need to represent the boundary of the city of Passau and fetch the current location of the vehicle. This requires modifying the XACML 3.0 data flow model in two parts:

1. A data type and function are needed to express geospatial properties (boundaries) and functionality within policies;
2. Means for obtaining the vehicle’s current location within the access control system must be established.

For the first part, we use the GeoXACML 3.0 [41] standard that extends the XACML specification with Geometry data type as well as a set of functions. The boundary lines of the city Passau can be easily represented via a *polygon*. Additionally, the vehicle can be represented as a data point using its coordinates—if it lies inside the polygon, then access to vehicle data is granted and if outside, then access is denied.

For the second part, to access the vehicle’s current location within the access control system by the PDP in order to evaluate location-specific authorization policies, an attribute provider is defined. The attribute provider gives the current GPS position of the vehicle as a data point, as shown in Listing 4 where the data point refers to the University of Passau, which is within Passau’s city limits.

Listing 4 **GeoXACML-Example Point Definition (University of Passau).**


<AttributeValue DataType="urn:ogc:def:dataType:geoxacml:1.0:geometry" geoxacml:crs="EPSG:4326">
    POINT (48.575 13.447)
</AttributeValue>

Linking the two parts together, it is now possible to implement an authorization policy (see Listing 5) that realizes the use case described in Section 4.3, which only allows access to vehicle location if the vehicle is located within the city limits of Passau.

Listing 5 **XACML-Location-Based Access Control Policy Example.**


<?xml version="1.0" encoding="UTF-8" standalone="no"?>
<Policy
  Version="1.0"
  PolicyId="LocationTest"
  xmlns="urn:oasis:names:tc:xacml:3.0:core:schema:wd-17"
  xmlns:geoxacml="http://www.opengis.net/geoxacml"
  RuleCombiningAlgId="urn:oasis:names:tc:xacml:3.0:rule-combining-algorithm:deny-unless-permit">
  <Target>
    <AnyOf>
      <AllOf>
        <Match
          MatchId="urn:oasis:names:tc:xacml:1.0:function:string-equal">
          <AttributeValue DataType="http://www.w3.org/2001/XMLSchema#string">instantshare</AttributeValue>
          <AttributeDesignator
            Category="urn:oasis:names:tc:xacml:1.0:subject-category:access-subject"
            AttributeId="urn:oasis:names:tc:xacml:1.0:subject:subject-id"
            MustBePresent="true"
            DataType="http://www.w3.org/2001/XMLSchema#string"/>
        </Match>
        <Match MatchId="urn:oasis:names:tc:xacml:1.0:function:string-equal">
          <AttributeValue
<!--Resource to be accessed-->
          DataType="http://www.w3.org/2001/XMLSchema#string">/vehicle/location</AttributeValue>
          <AttributeDesignator
            DataType="http://www.w3.org/2001/XMLSchema#string"
            AttributeId="urn:oasis:names:tc:xacml:1.0:resource:resource-id"
            Category="urn:oasis:names:tc:xacml:3.0:attribute-category:resource"
            MustBePresent="true"
          />
        </Match>
        <Match
          MatchId="urn:oasis:names:tc:xacml:1.0:function:string-equal">
<!--Action to be performed-->
          <AttributeValue DataType="http://www.w3.org/2001/XMLSchema#string">GET</AttributeValue>
          <AttributeDesignator
            DataType="http://www.w3.org/2001/XMLSchema#string"
            AttributeId="urn:oasis:names:tc:xacml:1.0:action:action-id"
            Category="urn:oasis:names:tc:xacml:3.0:attribute-category:action"
            MustBePresent="true"
          />
        </Match>
      </AllOf>
    </AnyOf>
  </Target>
  <Rule
    Effect="Permit"
    RuleId="PermitPassau">
    <Condition>
      <Apply
        FunctionId="urn:ogc:def:function:geoxacml:1.0:geometry-contains">
        <AttributeValue
          DataType="urn:ogc:def:dataType:geoxacml:1.0:geometry"
<!--City Passau boundaries defined using a Polygon-->
          geoxacml:crs="EPSG:4326">POLYGON ((
          48.574 13.479,
          48.577 13.462,
          48.574 13.438,
          48.575 13.431,
          48.574 13.421,
          48.577 13.412,
          48.560 13.400,
          48.552 13.435,
          48.558 13.438,
          48.572 13.463,
          48.574 13.479
          ))
        </AttributeValue>
        <Apply
<!--Function to obtain the current location of the vehicle---checks if the point is within or outside the Polygon-->
          FunctionId="urn:ogc:def:function:geoxacml:1.0:geometry-one-and-only">
          <AttributeDesignator
            DataType="urn:ogc:def:dataType:geoxacml:1.0:geometry"
            AttributeId="urn:tf:cyber:xacml:location:gpslocation"
            Category="urn:oasis:names:tc:xacml:3.0:attribute-category:environment"
            MustBePresent="true"
          />
        </Apply>
      </Apply>
    </Condition>
  </Rule>
</Policy>

#### 6.1.5. XACML4M Frequency-Based Access (FBAC)

In this requirement, to restrict access based on the frequency of requests, we need the following:

1. An access log mechanism to keep track of the frequency of access requests made by a subject;
2. A custom attribute provider for determining the last time a subject had accessed a resource.

We first highlight how access to resources by subjects is stored inside a log database and subsequently expound how this information can be used by the PDP for limiting access request frequency. We use an access log database implemented in the access-log-service component (see Figure 6). Two functionalities are provided by the access-log-service:

1. /save
2. /query

The first functionality, /save, is used to store successful access requests (after PDP permit) made inside the database, which is stored as triplets *subject, resource accessed, and action*. They are represented in JSON. An example access log entry is depicted in Listing 6.

Listing 6 **access-log-service-Log Entry (JSON).**


{
  "subject": "dashmon",
  "action": "GET",
  "resource": "/vehicle/camera"
}

Once an access log entry is sent to the logging service, it is assigned the current UNIX time in UTC at the resolution of milliseconds before being stored in the database. The second functionality, /query, can be used to fetch previous access time for a *subject* by analogously providing it with a combination of subject, action, and resource. As for our current example, the following response, as shown in Listing 7, is returned to /query:

Listing 7 **access-log-service-Log Query (JSON).**


{
  "subject": "dashmon",
  "action": "GET",
  "resource": "/vehicle/camera",
  "time": 1649964600000
}

In case the resource is accessed for the *first time* through the subject via some action, i.e., no log entry is present in the database, the time property is automatically set to 0. For submitting accesses to the log service, XACML obligations as previously introduced in Section 6.1.1 are utilized. This implies that accesses are not logged via the PDP, but through the *Obligations Service* that has been implemented as part of the *PEP*.

The corresponding obligation for logging accesses to access-log-service was named LogObligation. The key advantage of this approach is that logging may be enabled selectively as part of defining XACML policies, resulting in potentially saving disk space and, in some cases, even avoiding privacy issues that arise with logging. Logging can be enabled by means of <ObligationExpression> blocks on both *policies* as well as *rule* levels and require the specification of three attributes through <AttributeAssignmentExpression> tags. These must specify the *subject, action, and resource* belonging to the access request to be logged. Its usage is depicted in Listing 8.

Listing 8 **XACML-LogObligation Obligation Usage—to enable logging after PDP grants access to the Subject.**


<ObligationExpressions>
<!--Obligation requires 3 attributes for logging-->
  <ObligationExpression ObligationId="LogObligation" FulfillOn="Permit">
<!--Attribute 1: Subject requesting access-->
    <AttributeAssignmentExpression AttributeId="LogObligation:Subject">
      <AttributeDesignator
        MustBePresent="true"
        Category="urn:oasis:names:tc:xacml:1.0:subject-category:access-subject"
        AttributeId="urn:oasis:names:tc:xacml:1.0:subject:subject-id"
        DataType="http://www.w3.org/2001/XMLSchema#string" />
    </AttributeAssignmentExpression>
<!--Attribute 2: Action performed by the Subject-->
    <AttributeAssignmentExpression AttributeId="LogObligation:Action">
      <AttributeDesignator
        MustBePresent="true"
        Category="urn:oasis:names:tc:xacml:3.0:attribute-category:action"
        AttributeId="urn:oasis:names:tc:xacml:1.0:action:action-id"
        DataType="http://www.w3.org/2001/XMLSchema#string" />
    </AttributeAssignmentExpression>
<!--Attribute 3: Resource accessed by the Subject-->
    <AttributeAssignmentExpression AttributeId="LogObligation:Resource">
      <AttributeDesignator
        MustBePresent="true"
        Category="urn:oasis:names:tc:xacml:3.0:attribute-category:resource"
        AttributeId="urn:oasis:names:tc:xacml:1.0:resource:resource-id"
        DataType="http://www.w3.org/2001/XMLSchema#string" />
    </AttributeAssignmentExpression>
  </ObligationExpression>
</ObligationExpressions>

If a rule or policy specifying the <ObligationExpression> above comes into effect, then the PDP after evaluating such policy would specify LogObligation, *subject, resource, and action* in its response to the PEP. Thus, it informs the PEP to log the access made by the subject. The current PEP component only supports this single LogObligation and efforts have been made to make new Obligation extensions as easy as possible in XACML4M.

Additionally, since the time information on previous accesses is available in access-log-service, the *Polling Frequency Provider* component in Figure 6 provides three different attributes for the PDP that interfaces with the database. They are:

1. polling-frequency:time-since-last-access:ms: Returns the time difference since the resource has been accessed by the subject via some action in *milliseconds*.
2. polling-frequency:time-since-last-access:s: Returns the time difference since the resource has been accessed by the subject via some action, giving lower time resolution in *seconds*.
3. polling-frequency:hz: Provides the access frequency if the resource were accessed at the current point in time, depending on the previous access time (i.e., the inverse of the last access time delta to the current time).

Finally, we have all the components necessary to meet the *Frequency-Based Access Control* requirement. The use case previously introduced in Section 4.4, where camera data shall only be accessed every 33 milliseconds, can be implemented using the following rule which simply applies a numerical comparison operator on the access time delta shown in Listing 9.

Listing 9 **XACML-Polling Frequency (Milliseconds).**


<Rule Effect="Permit" RuleId="PollingFrequencySample">
  <Condition>
    <Apply FunctionId="urn:oasis:names:tc:xacml:1.0:function:integer-greater-than">
      <Apply FunctionId="urn:oasis:names:tc:xacml:1.0:function:integer-one-and-only">
        <AttributeDesignator
          DataType="http://www.w3.org/2001/XMLSchema#integer"
<!--Fetches time difference between last and current access in ms-->
          AttributeId="urn:tf:cyber:xacml:polling-frequency:time-since-last-access:ms"
          Category="urn:oasis:names:tc:xacml:3.0:attribute-category:environment"
          MustBePresent="true" />
      </Apply>
      <AttributeValue
<!--Defining the frequency of access-->
      DataType="http://www.w3.org/2001/XMLSchema#integer">33</AttributeValue>
    </Apply>
    </Condition>
</Rule>

To summarize, all identified requirements specific to automotive access control are fully realized in our XACML4M data flow model (based on XACML) by methodically adding components specific to each requirement (see Figure 6). Our implementation of the proposed XACML4M is accessible at this link [42], which uses *AuthzForce PDP* (an XACML PDP implementation) [29].

Thus, we can now show that ABAC-based XACML can be extended to include *signal access, location, time, and frequency* requirements of connected vehicles, thus partially answering the RQ 1. Furthermore, RQ 1 is still open because XACML4M has only been developed and not tested, and to verify that it functionally satisfies these requirements, we implement a proof of concept of the exemplary use case (see Section 3) and evaluate it for functionality based on authorization policies.

## 7. Evaluation

For functional evaluation of our XACML4M data flow model, we implement in Java a prototype based on our requirements—*Signal Access, Time-Based Access, Location-Based Access, and Frequency-Based Access*. Then, to verify the XACML4M data flow model, we perform the functional evaluation based on the authorization policies.

In this paper, we focus only on the authorization of an application/service to access vehicle data. We assume that all requesting applications/services are already authenticated into the system.

### 7.1. Experimental Setup

An ARM-based Raspberry Pi 4 development board running Ubuntu Linux was chosen as the target platform for all subsequent experiments. A summary of its specifications [43] are listed in Table 2. The Raspberry Pi 4 was chosen because its CPU performance is similarly constrained to x86-based Intel Atom chips that are used within existing infotainment systems.

Concerning the vehicle, a *Peugeot 108 compact car* serves as the data source. We do not directly access vehicle sensor data from the ECU because it requires low-level access via Controller Area Network (CAN) bus. Physically tapping into the vehicle’s bus systems is certainly possible, but this was not taken into further consideration due to legal and, primarily, safety concerns. Instead, we used the OBD-II port of the *Peugeot 108 compact car* to access diagnostic sensor data. OBD-II port is chosen because it is present in virtually every vehicle today as a result of an EU directive in the year 1998, which mandates it to be included in every car sold within the bounds of the EU from 2001 (for petrol engines) and 2003 (for diesel engines) [44].

A *Vehicle Data Environment (VDE)* is an environmental setup developed using HTTP REST service for data access from inside the vehicle via the OBD-II port. OBD-II provides different services by accessing the CAN bus, such as reading sensor data, fetching and clearing diagnostic trouble codes, and querying the vehicle for diagnostic test results, among others. For our experiments, we only need to access the *reading sensor data* service. VDE was necessary because of our limitation that there was no collaboration with a vehicle manufacturer and most of the tools, data simulators, and runtime environments were proprietary [45,46].

The data provided by the VDE are actual real-time data and the pre-requisite is that the vehicle engine is running for data to be retrieved via the OBD-II port. Due to the prevailing limitation, only sensor data could be collected. Actuators and ECU-related data were not accessible, although this has no effect on the resulting access control implementation. The values retrieved from the OBD-II port of the vehicle are hexadecimal numbers and SAE J1979/ISO 15031-5 provides a formula for interpreting different sensor data and converting them into decimal values.

Different sensor data could be retrieved from the vehicle but for this paper, we only consider *location*, *engineSpeed*, *vehiclesspeed* depending on the use case.

### 7.2. Functional Evaluation

In this section, the functionality and correctness of the implementation are examined and illustrated by evaluating the use case presented in Section 3. To recap, the key conditions which also illustrate the requirements under which Alice wants to share her vehicle sensor data with *SmartSurance* service are:

- Signal Access: Only *current location, vehicle speed, engine RPM, and throttle position* sensor data can be shared to *SmartSurance*.
- Location-Based Access: Vehicle data access is granted only when she travels outside her town of Haiden, Bavaria, Germany.
- Time-Based Access: Vehicle data access is denied for weekdays between *5:05 p.m.* and *8:00 p.m.*
- Frequency-Based Access: *SmartSurance* requires one sample of the sensor values per second.

These conditions are specified in the authorization policies and are presented for each functional evaluation below. Since the XML policies are verbose and they have already been explained in Section 6, only important *conditions* are shown in the policies below.

#### 7.2.1. Location-Based Access Control

The initial setting for the functional test is as below: The vehicle is parked just outside the town of Haiden as its initial position. The boundaries of the town Haiden are defined with a polygon that is specified in the authorization policy (as was done for Section 6.1.4). See Listing 10 for LBAC-specific authorization policy.

Listing 10 **XACML4M-Location Based Access Policy.**


<Policy PolicyId="SmartSuranceWrongLocation"
        <Target></Target>
        <Rule RuleId="DenyWrongPosition" Effect="Deny">
            <Description>Denies all incoming requests by SmartSurance when vehicle is inside hometown.</Description>
            <Condition>
!--Function to check if the current vehicle location is inside the defined polygon of the city of Haiden -->
                <Apply FunctionId="urn:ogc:def:function:geoxacml:1.0:geometry-contains">
                    </AttributeValue>
                    <Apply FunctionId="urn:ogc:def:function:geoxacml:1.0:geometry-one-and-only">
                        <AttributeDesignator
                        DataType="urn:ogc:def:dataType:geoxacml:1.0:geometry"
!--Access to Location Data-->
                        AttributeId="urn:tf:cyber:xacml:location:gpslocation"
                        Category="urn:oasis:names:tc:xacml:3.0:attribute-category:environment"
                        MustBePresent="true"
                        />
                    </Apply>
                </Apply>
            </Condition>
        </Rule>
    </Policy>

**Figure 8:**
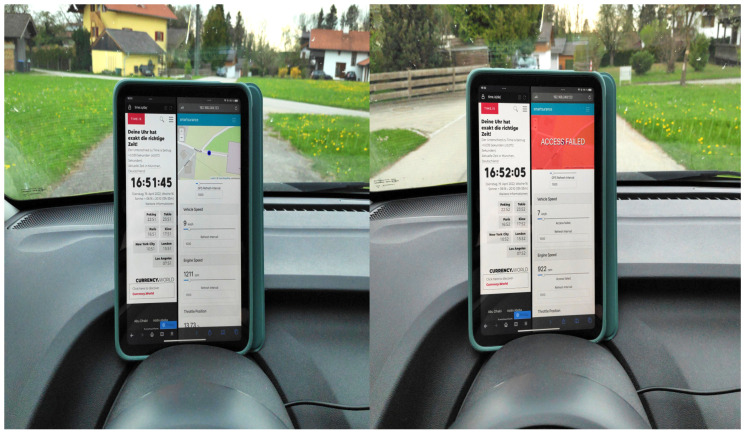
Functional Evaluation—Location-based Access Control (access denied when inside the town of Haiden, as defined by a polygon) tested on the SmartSurance Application.

The policy grants/denies access to vehicle sensor data based on its location, i.e., as per our use case, if the vehicle is in Haiden, then access is denied, otherwise, it is granted. The results of this experiment for location-based access control (LBAC) are depicted in Figure 8, which was conducted around 4:51 p.m.

When the vehicle is in its initial position, i.e., outside the defined boundary in the policy, then access is granted to *location*, data as shown in the left half of Figure 8. Then, the vehicle moves and enters the boundary marked by the polygon, i.e., in Haiden, and thus, access is denied accordingly, as shown in the right half of Figure 8. Thus, the functionality of LBAC in XACML4M is verified.

#### 7.2.2. Time-Based Access Control

**Figure 9:**
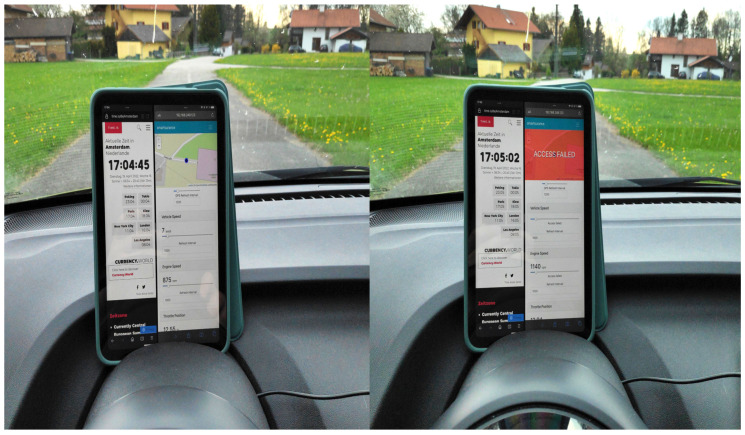
Functional Evaluation—Time-based Access Control (access denied between 05:05 PM–8:00 PM) tested on the SmartSurance Application.

Next, the functionality of time-based access control was examined in a subsequent second experiment, where the vehicle was moved back to its initial position, just outside of Haiden. The TBAC-based policy is listed in Listing 11. The first access request is made before *5:05 p.m.* and it is granted because it meets the authorization policies condition (see left part of Figure 9). The second request is made after *5:05 p.m.* and it violates the policy condition and subsequently, access is denied (see right part of Figure 9). Thus, the functionality of TBAC in XACML4M is verified.

Listing 11 **XACML-Time Based Access Policy (Time in Milliseconds).**


<Policy PolicyId="SmartSuranceOffPeakHours"
        <Target></Target>
        <Rule RuleId="DenyOffHours" Effect="Deny">
            <Description>Denies all incoming requests by SmartSurance during off-peak hours.</Description>
            <Condition>
                <Apply FunctionId="urn:oasis:names:tc:xacml:1.0:function:and">
                    <Apply FunctionId="urn:oasis:names:tc:xacml:3.0:function:dateTime-in-dayOfWeek-range">
                        <Apply FunctionId="urn:oasis:names:tc:xacml:1.0:function:dateTime-one-and-only">
                            <AttributeDesignator
                            Category="urn:oasis:names:tc:xacml:3.0:attribute-category:environment"
                            AttributeId="urn:oasis:names:tc:xacml:1.0:environment:current-dateTime"
                            DataType="http://www.w3.org/2001/XMLSchema#dateTime"
                            MustBePresent="false"/>
                        </Apply>
<!--Monday (1), Tuesday (2)... Friday(5)---below, we define the day range from Monday to Friday when access is denied-->
                        <AttributeValue DataType="urn:oasis:names:tc:xacml:3.0:data-type:dayOfWeek">1+02:00</AttributeValue>
                        <AttributeValue DataType="urn:oasis:names:tc:xacml:3.0:data-type:dayOfWeek">5+02:00</AttributeValue>
                    </Apply>
                    <Apply FunctionId="urn:oasis:names:tc:xacml:2.0:function:time-in-range">
                        <Apply FunctionId="urn:oasis:names:tc:xacml:1.0:function:time-one-and-only">
                            <AttributeDesignator
                            DataType="http://www.w3.org/2001/XMLSchema#time"
                            AttributeId="urn:oasis:names:tc:xacml:1.0:environment:current-time"
                            Category="urn:oasis:names:tc:xacml:3.0:attribute-category:environment"
                            MustBePresent="true"/>
                        </Apply>
<!--Time Limit when access is denied-->
                        <AttributeValue DataType="http://www.w3.org/2001/XMLSchema#time">17:05:00+02:00</AttributeValue>
                        <AttributeValue DataType="http://www.w3.org/2001/XMLSchema#time">20:00:00+02:00</AttributeValue>
                    </Apply>
                </Apply>
            </Condition>
        </Rule>
    </Policy>

#### 7.2.3. Frequency-Based Access Control

**Figure 10:**
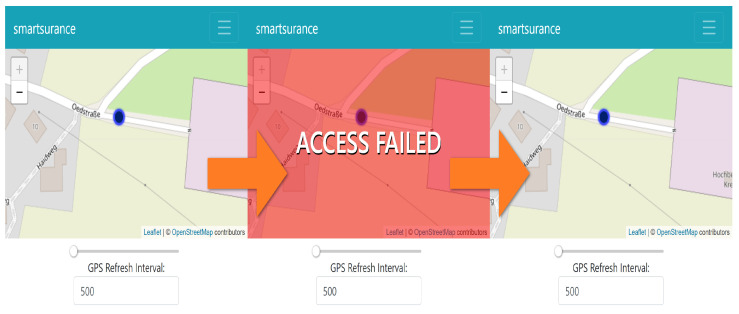
Functional Evaluation—Frequency-Based Access (access granted every 1000 ms) tested on the SmartSurance Application [47].

For verifying the frequency-based access control requirement, the *SmartSurance* application has sliders that change the *frequency* of access (see Figure 10) made by *SmartSurance*. In our use case Section 3, we defined the access frequency *condition* to be 1 data point per second, which amounts to 1 data point every *1000 ms*. Listing 12 shows the corresponding authorization policy for the use case, which is then evaluated by the PDP in XACML4M.

Listing 12 **XACML4M—Frequency Based Access Policy.**


    <Policy PolicyId="SmartSuranceSensorData"
        <Target>
                    <Match MatchId="urn:oasis:names:tc:xacml:1.0:function:string-equal">
                        <AttributeValue DataType="http://www.w3.org/2001/XMLSchema#string">GET</AttributeValue>
                        <AttributeDesignator DataType="http://www.w3.org/2001/XMLSchema#string"
                             AttributeId="urn:oasis:names:tc:xacml:1.0:action:action-id"
                             Category="urn:oasis:names:tc:xacml:3.0:attribute-category:action"
                             MustBePresent="true"
                        />
                    </Match>
        </Target>
        <Rule
                Effect="Permit"
                RuleId="VehicleLocation">
            <Description>Allows access to vehicle location data every 1000 milliseconds only.</Description>
            <Target>
                        <Match MatchId="urn:oasis:names:tc:xacml:1.0:function:string-equal">
                            <AttributeValue DataType="http://www.w3.org/2001/XMLSchema#string">/vehicle/location</AttributeValue>
                            <AttributeDesignator
                                Category="urn:oasis:names:tc:xacml:3.0:attribute-category:resource"
                                AttributeId="urn:oasis:names:tc:xacml:1.0:resource:resource-id"
                                MustBePresent="true"
                                DataType="http://www.w3.org/2001/XMLSchema#string"/>
                        </Match>
            </Target>
            <Condition>
<!--Checking with the Access Log Database and comparing last successful access with the current request-->
                <Apply FunctionId="urn:oasis:names:tc:xacml:1.0:function:integer-greater-than-or-equal">
                    <Apply FunctionId="urn:oasis:names:tc:xacml:1.0:function:integer-one-and-only">
                        <AttributeDesignator DataType="http://www.w3.org/2001/XMLSchema#integer"
                         AttributeId="urn:tf:cyber:xacml:polling-frequency:time-since-last-access:ms"
                         Category="urn:oasis:names:tc:xacml:3.0:attribute-category:environment"
                         MustBePresent="true"
                        />
                    </Apply>
                    <AttributeValue DataType="http://www.w3.org/2001/XMLSchema#integer">1000</AttributeValue>
                </Apply>
            </Condition>
        </Rule>

As part of this experiment, the access frequency made by *SmartSurance* application was set to 500 ms (to test for failed cases), thus violating the minimum *condition* of 1000 ms, as specified in the policy. Figure 10 depicts three consecutive requests for location data. Only the second access request, whose response is highlighted in *red*, is denied because the access request was made in less than 1000 ms. By the time the third access request was received, the condition of 1000 ms in the policy was satisfied and thus, access to *location* data point was granted based on frequency. Thus, the functionality of FBAC in XACML4M was verified.

So far, we have evaluated each identified automotive access control requirement in XACML4M based on authorization policies and can validate the functionality.

### 7.3. Discussion

To summarize, in this section, we have tested the XACML4M framework for functionality with regard to signal access, location-based access, time-based access, and frequency-based access requirements. The XACML4M data flow model was verified by implementing authorization policies derived from our use case. Table 3 gives an overview of the results of the functional evaluation for XACML4M.

Our results lead to the following conclusions:

- Functional evaluation based on authorization policies for the defined use case was valid because access to vehicle sensor data was granted or denied only after policy evaluation;
- ABAC-based XACML access control model with additional components (XACML4M) can satisfy signal access, time-based access, location-based access, and frequency-based access requirements in automotive.

Note that the *signal access* requirement is implicitly tested in the evaluation of each of the above more complex requirements and is considered fulfilled. The results help answer the research question 1 that an ABAC-based XACML can be modified to meet the automotive domain’s requirements. In addition, XACML4M is a flexible data flow model based on extensible XACML, implying that future automotive-specific access control requirements such as resource consumption restrictions on an application can also be potentially integrated.

Comparing the result and referring to our brief literature overview in Table 1,^26^,^27^,^28^] consider at most three of our requirements. Although the requirements are mentioned briefly in each, no further information is available, either because the project was disbanded [26], or because there was no exemplary policy implementation to compare with. This implies that a thorough literature review is required and additional evaluation based on the performance of our proposed *XACML4M* model is necessary.

Consequently, this brings us to the limitations of our work. The XACML4M framework was installed on a Raspberry Pi and not a real vehicle. This could introduce potential limitations on the available resources and processing power for XACML4M running on the Raspberry Pi when compared to a real vehicle, even if Raspberry Pi has similar hardware constraints as the infotainment unit inside a vehicle.

Next, only a limited number of policies were defined, typically with a maximum of up to three rules within a policy. This limits the scope of the evaluation, which would require experimenting with more complex and real-life scenarios, not only to evaluate the functionality, but also the prowess of the access control algorithm, its performance, and its limitations.

## 8. Conclusions and Future Work

In a V2X setting, vehicles are complex systems with a multitude of sensors that generate and share data with their surroundings. Such a connected vehicle ecosystem requires a distributed and fine-grained access control mechanism to protect vulnerable data for security and safety reasons. The challenge in designing such a system for connected vehicles (CVs) comes from correctly assessing the vulnerability of a vehicle’s data in the context of its constantly changing environment. For instance, a vehicle owner who wishes to share their vehicle data on weekdays for traffic management might not want to do so on weekends or when traveling outside of their local city. This led us to define a real-world use case, identify requirements specific to connected vehicles, i.e., *signal access, time-based access, location-based access, and frequency-based access*, and investigate our research question mentioned below:

*RQ: How can Attribute-Based Access Control (ABAC) fulfill connected vehicle requirements of signal access, time-based access, location-based access, and frequency-based access control?*

To answer this *RQ*, this paper proposes XACML4M—an XACML-based data flow model that was methodically developed to satisfy all four requirements. The following components were added to the data flow model of XACML:

- *Vehicle Data Environment (VDE) integrated with Policy Enforcement Point (PEP)*: PEP first receives all incoming access requests from applications and services, while VDE enables vehicle sensor data to be accessed.
- *Time Extensions*: Provide *time period* data type as an attribute for policy evaluation by PDP.
- *GeoLocation Provider*: Provides *geospatial* data type as an attribute and also retrieves the current location of the vehicle for policy evaluation by the PDP.
- *Polling Frequency Provider*: To fetch the timestamp of the last access made by a requesting entity, e.g., an application or a service.
- *Access Log Service*: To log the last successful access made by a requesting entity, e.g., an application or a service.

To test the developed XACML4M data flow model’s functionality against our four main requirements, a proof of concept was implemented based on a real-world use case. Based on a successful functional evaluation using authorization policies created specifically for the use case, it was concluded that XACML4M can meet the identified requirements for connected vehicles. Thus, our *RQ* 1 is answered.

Our proposed XACML4M data flow model provides an access control mechanism that considers time, location, and frequency of access within a single framework for connected vehicles. There is still some scope for future work based on our limitations. First, we plan to extend the evaluation by considering performance metrics such as computation time taken by each component in the data flow model, maximum request load, policy evaluation time based on different policy sizes, and service time for a request.

Measuring the time required for message flow between various components could be one method of achieving this. In particular, for use cases with soft real time requirements, this could help identify bottlenecks within the framework and optimize it for quicker policy evaluation. Additionally, different versions of XACML4M could be developed based on these performance metrics and help evaluate the usability of the framework.

Second, the introduction of GDPR has raised complex issues on data subjects’ rights, e.g., right to information (Article 13), right to erase personal data (Article 17), and right to access personal data (Article 15). Purpose-based access control mechanisms should also be considered given that the vehicle sensor data contain personal information, such as the location of the vehicle owner and travel route. We plan to add *Purpose* to the access control system of XACML4M to make it future-proof.

Third, to comply with GDPR specifications, the current use case and, thus, the policies, would become complex, requiring GDPR-specific attribute providers, data transformation requirements, and different action restrictions. As a result, a visual specification language is required to represent and translate policies into XACML [32,33,34,35,37,38]. We intend to develop a visual specification language on top of XACML for the creation and maintenance of the policies.

Next, an argument can be made that the implemented functionality can also be realized using an alternate policy language such as ALFA [31] instead of XACML. Therefore, alternative implementations of our functionality with such policy languages would have to be evaluated and compared to our proposed model.

Last but not least, one of the limitations of our research is the lack of access to a real vehicle in which we could set up and test our framework. It is important to monitor and understand the effects of request load and the effectiveness of our access control mechanism in a realistic automotive setting.

## Figures and Tables

**Table 1:** Requirement Fulfillment Status for Signal Access Control (SAC), Frequency-Based Access Control (FBAC), Time-Based Access Control (TBAC), and Location-Based Access Control (LBAC).

Literature	SAC	FBAC	TBAC	LBAC	Access Control Model
Kim et al. [7]	✓	×	×	×	RBAC
Gupta et al. [6]	✓	×	×	✓	ABAC
Rumez et al. [5]	✓	✓	×	×	ABAC
Kim et al. [8]	✓	✓	×	×	ABAC
Plappert et al. [26]	✓	✓	×	✓	ABAC
Zhang et al. [27]	✓	×	✓	✓	DAC, IBAC, ABAC
Alsarra et al. [28]	✓	×	✓	✓	OpenRBAC
Albouq et al. [19]	✓	×	×	×	ABAC
Kchaou et al. [24]	×	×	×	×	ABAC

**Table 2:** Experimental Setup—Hardware Overview.

**Board**	Raspberry Pi 4
**CPU**	Broadcom BCM2711 (ARMv8), 4x 1.5GHz (performance governor)
**Memory**	4GB LPDDR4 @ 3200MHz
**Storage**	Netac 32GB microSD (UHS-I)
**OS**	Ubuntu 22.04 (arm64)
**JVM**	OpenJDK JRE 11
**GPS**	VK-162, UBX-G7020 chipset
**OBD-II**	Veepak OBD-II Adapter (ELM327 clone-based)
**Vehicle**	Peugeot 108 compact car

**Table 3:** XACML4M—Functionality Evaluation Overview (✓ indicates that the functionality was satisfied).

Requirement	XACML4M
Signal Access	✓
Location-Based Access	✓
Time-Based Access	✓
Frequency-Based Access	✓

## Data Availability

The data presented in this study are available on request from the corresponding author.

## References

[1] Collingwood L. (2017). Privacy implications and liability issues of autonomous vehicles. Inf. Commun. Technol. Law.

[2] Miller C., Valasek C. Remote exploitation of an unaltered passenger vehicle. Proceedings of the Black Hat USA.

[3] Pesé M.D., Shin K.G. (2019). Survey of Automotive Privacy Regulations and Privacy-Related Attacks.

[4] Krontiris I., Grammenou K., Terzidou K., Zacharopoulou M., Tsikintikou M., Baladima F., Sakellari C., Kaouras K. (2020). Autonomous Vehicles: Data Protection and Ethical Considerations. Proceedings of the Computer Science in Cars Symposium, CSCS ’20.

[5] Rumez M., Duda A., Gründer P., Kriesten R., Sax E. Integration of Attribute-based Access Control into Automotive Architectures. Proceedings of the 2019 IEEE Intelligent Vehicles Symposium (IV).

[6] Gupta M., Benson J., Patwa F., Sandhu R. (2019). Dynamic Groups and Attribute-Based Access Control for Next-Generation Smart Cars. Proceedings of the Ninth ACM Conference on Data and Application Security and Privacy, CODASPY ’19.

[7] Kim D., Ju H., Jung B., Na J.C. An Access Control Method for Vehicle Management System. Proceedings of the 9th International Conference on Information and Communication Technology Convergence: ICT Convergence Powered by Smart Intelligence, ICTC 2018.

[8] Kim D.K., Song E., Yu H. (2016). Introducing Attribute-Based Access Control to AUTOSAR.

[9] Coppola P., Silvestri F., Coppola P., Esztergár-Kiss D. (2019). Autonomous vehicles and future mobility solutions. Autonomous Vehicles and Future Mobility.

[10] Fleming W. (2008). New Automotive Sensors—A Review. Sensors.

[11] Tyler N. (2016). Safe and Secure. https://assets.markallengroup.com//article-images/149323/P24-25.pdf.

[12] Siegel J.E., Erb D.C., Sarma S.E. (2018). A Survey of the Connected Vehicle Landscape—Architectures, Enabling Technologies, Applications, and Development Areas. IEEE Trans. Intell. Transp. Syst..

[13] Ahangar M.N., Ahmed Q.Z., Khan F.A., Hafeez M. (2021). A Survey of Autonomous Vehicles: Enabling Communication Technologies and Challenges. Sensors.

[14] Roddeck W. (1997). Aktoren. Einführung in die Mechatronik.

[15] Le V.H., Hartog J., Zannone N. (2018). Security and privacy for innovative automotive applications: A survey. Comput. Commun..

[16] Nolte T., Hansson H., Lo Bello L. Automotive communications-past, current and future. Proceedings of the 2005 IEEE Conference on Emerging Technologies and Factory Automation.

[17] (2016). Requirements for the application of ECUs in e-mobility originally qualified for gasoline cars. Microelectron. Reliab..

[18] Sommer C., Dressler F. (2014). Vehicular Networking.

[19] Albouq S.S., Fredericks E.M. Securing communication between service providers and road side units in a connected vehicle infrastructure. Proceedings of the 2017 IEEE 16th International Symposium on Network Computing and Applications, NCA 2017.

[20] Hu V., Ferraiolo D., Kuhn D., Schnitzer A., Sandlin K., Miller R., Scarfone K. (2014). Guide to Attribute Based access Control (ABAC) Definition and Considerations.

[21] eXtensible Access Control Markup Language (XACML) Version 3.0 22 January 2013. OASIS Standard..

[22] W3C (2020). W3C Automotive Working Group. https://www.w3.org/groups/wg/auto.

[23] Ashutosh A., Gerl A. Access Control for a Connected Vehicle Ecosystem. Proceedings of the 2021 11th International Conference on Advanced Computer Information Technologies, ACIT 2021-Proceedings.

[24] Kchaou A., Ayed S., Abassi R., Fatmi S.G.E. Smart Contract-Based Access Control for the Vehicular Networks. Proceedings of the 2020 International Conference on Software, Telecommunications and Computer Networks (SoftCOM).

[25] (2021). Layered Software Architecture. https://www.autosar.org/fileadmin/user_upload/standards/classic/21-11/AUTOSAR_EXP_LayeredSoftwareArchitecture.pdf.

[26] Plappert C., Zelle D., Krauß C., Lange B., Mauthöfer S., Walter J., Abendroth B., Robrahn R., von Pape T., Decke H. A privacy-aware data access system for automotive applications. Proceedings of the 15th ESCAR Embedded Security in Cars Conference.

[27] Zhang Q., Zhong H., Cui J., Ren L., Shi W. (2021). AC4AV: A Flexible and Dynamic Access Control Framework for Connected and Autonomous Vehicles. IEEE Internet Things J..

[28] Alsarra S., Yen I.L., Huang Y., Bastani F., Thuraisingham B. (2019). An OpenRBAC Semantic Model for Access Control in Vehicular Networks. Proceedings of the 24th ACM Symposium on Access Control Models and Technologies, SACMAT ’19.

[29] OW2 AuthzForce Community Edition. https://github.com/authzforce.

[30] WSO2 wso2/balana. https://github.com/wso2/balana.

[31] (2015). Abbreviated Language for Authorization Version 1.0. https://www.oasis-open.org/committees/download.php/55228/alfa-for-xacml-v1.0-wd01.doc.

[32] Ouaddah A., Mousannif H., Abou Elkalam A., Ait Ouahman A. (2017). Access control in the Internet of Things: Big challenges and new opportunities. Comput. Netw..

[33] Heydon A., Maimone M., Tygar J., Wing J., Zaremski A. (1990). Miro: visual specification of security. IEEE Trans. Softw. Eng..

[34] Giordano M., Polese G. (2013). Visual Computer-Managed Security: A Framework for Developing Access Control in Enterprise Applications. IEEE Softw..

[35] Basin D., Doser J., Lodderstedt T. (2006). Model Driven Security: From UML Models to Access Control Infrastructures. ACM Trans. Softw. Eng. Methodol..

[36] Koch M., Mancini L.V., Parisi-Presicce F. (2002). A Graph-Based Formalism for RBAC. ACM Trans. Inf. Syst. Secur..

[37] Nergaard H., Ulltveit-Moe N., Terje G. A scratch-based graphical policy editor for XACML. Proceedings of the 2015 International Conference on Information Systems Security and Privacy (ICISSP).

[38] Gerl A., Meier B., Becher S., Ahram T., Taiar R., Colson S., Choplin A. (2020). Let Users Control Their Data—Privacy Policy-Based User Interface Design. Proceedings of the Human Interaction and Emerging Technologies.

[39] (2017). JSON Profile of XACML 3.0 Version 1.0. https://docs.oasis-open.org/xacml/xacml-json-http/v1.0/xacml-json-http-v1.0.html.

[40] (2019). XACML v3.0 Time Extensions Version 1.0. https://docs.oasis-open.org/xacml/xacml-3.0-time-extensions/v1.0/csprd01/xacml-3.0-time-extensions-v1.0-csprd01.html.

[41] (2013). OGC Geospatial eXensible Access Control Markup Language (GeoXACML) 3.0 Core. https://portal.opengeospatial.org/files/?artifact_id=55231.

[42] XACML4M XACML4M Implementation. https://gitlab.com/simonwagner/automotive-access-control/.

[43] Foundation R.P. Raspberry Pi 4 Tech Specs. https://www.raspberrypi.com/products/raspberry-pi-4-model-b/specifications/.

[44] DIRECTIVE 98/69/EC (1998). European Union. https://eur-lex.europa.eu/LexUriServ/LexUriServ.do?uri=CONSLEG:1998L0069:19981228:EN:PDF.

[45] Limited Q.S.S. BlackBerry QNX Automotive Software for Connected and Autonomous Vehicles. https://blackberry.qnx.com/en/industries/connected-autonomous-vehicles.

[46] GmbH V.I. MICROSAR Classic-Die Intelligente Implementierung des AUTOSAR Classic-Standards. https://www.vector.com/de/de/produkte/produkte-a-z/embedded-components/microsar/.

[47] (2017). OpenStreetMap Contributors. https://www.openstreetmap.org.

